# Hydrogen peroxide, ascorbate, and glutathione: building the Foyer–Halliwell–Asada pathway

**DOI:** 10.1007/s00425-025-04702-4

**Published:** 2025-05-08

**Authors:** Graham Noctor

**Affiliations:** 1 Institut Des Sciences des Plantes de Paris-Saclay, Unité Mixte de Recherche 8618 Centre National de La Recherche Scientifique, Université de Paris-Sud, 91405 Orsay Cedex, France; 2 Institut Universitaire de France (IUF), Paris, France

**Keywords:** Antioxidant, Glutathione reductase, Hydrogen peroxide, Peroxidase, Reactive oxygen species, Redox

## Abstract

Ascorbate and glutathione are water-soluble compounds that are found at high concentrations in many plant tissues. A close association between the two molecules has been noted almost since the time Planta was founded, 100 years ago. Although both have many functions, much attention has been paid to their influence as antioxidants. One of the conceptual turning-points regarding the significance of these compounds in plants occurred in the second half of the 1970s, when the ascorbate–glutathione pathway was first characterized as a chloroplastic antioxidative process. Now known as the Foyer–Halliwell–Asada pathway, this sequence of reactions notably links reduction of H_2_O_2_, catalysed by ascorbate peroxidase, to oxidation of NADH or NADPH, catalysed by monodehydroascorbate reductase and glutathione reductase. One of the papers that laid the foundation stones of the pathway was Foyer and Halliwell (Planta 133:21–25, 1976). This perspective takes a look back at the contributions of this and related work in the context of plant biology research at the time, and considers the importance of the pathway within our current understanding of reactive oxygen species biology and redox homeostasis and signalling. Emphasis is placed on the advances in our knowledge of the genes and proteins involved and the potential metabolic flexibility of the pathway, as well as its place within the highly intricate plant network of H_2_O_2_-metabolising systems.

## Introduction

Biological evolution can involve the adaptation of existing structures to new functions. In a similar way, scientific understanding often advances in unforeseen directions, with the application of available knowledge to new contexts. The development of our concepts of antioxidant metabolism in plants offers a striking example of this process. By the middle of the twentieth century, the idea of redox coupling between ascorbate and glutathione had been proposed by several researchers. A ‘close association between ascorbate and glutathione in plant tissues’ was noted in the 1950s (Mapson and Goddard 1951). In fact, it had been documented more than ten years earlier that ‘when ascorbic acid and glutathione are together in the presence of the hexoxidase [ascorbate oxidase] described by Szent-Gyorgyi the glutathione wholly protects the vitamin from oxidation, whilst it is itself oxidized at a rate which, with the same concentration of enzyme, is exactly the same as the rate with which ascorbic acid is oxidized when alone.’ (Hopkins and Morgan 1938). By the middle of the twentieth century, enzymatic systems able to catalyse NADPH-dependent reduction of glutathione had also been detected in plant tissues (Conn and Vennesland 1951; Mapson and Goddard 1951).

**Fig. 1:**
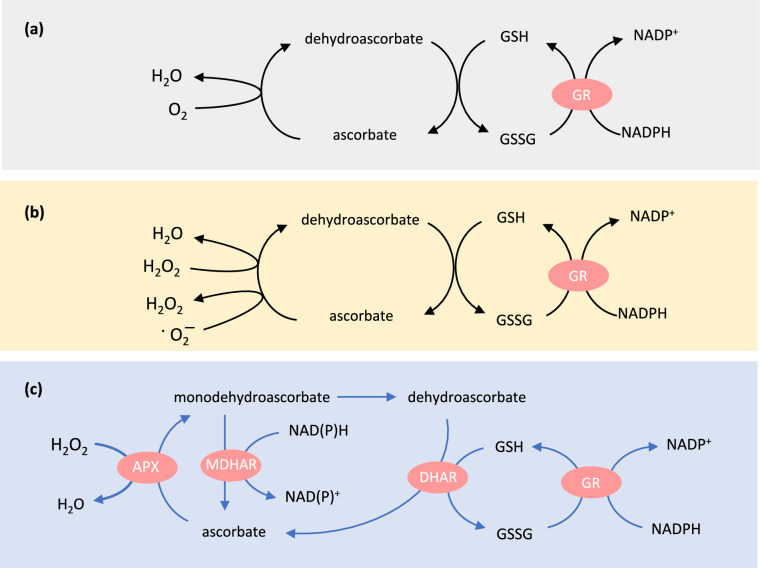
The ascorbate–glutathione pathway, then and now. **a** Oxygen-dependent respiratory chain proposed to operate in pea seeds by Mapson and Goddard (1951). **b** The ROS-linked chloroplast pathway of Foyer and Halliwell (1976). **c** An updated form that includes the activities of ascorbate peroxidase (APX), monodehydroascorbate reductase (MDHAR), and dehydroascorbate reductase (DHAR). Although part **c** focuses on enzyme-dependent reduction of H_2_O_2_, other ROS such as superoxide can chemically oxidize both ascorbate and glutathione. Reactions are not shown stoichiometrically. GR, glutathione reductase. GSH, reduced glutathione. GSSG, glutathione disulfide

At the time of these studies, the roles of reactive oxygen species (ROS) in cells were poorly characterised and information on subcellular compartmentation was at best fragmentary. Generation of H_2_O_2_ by mitochondria and chloroplasts had been reported by Mehler (1951) but it was assumed that H_2_O_2_ formation from O_2_ was direct and that the molecule would be removed by enzymes such as catalases, which are now not considered to be found in these organelles. The nature of the ascorbate-oxidizing routes was not clear. Although the possibility of a peroxidase-based reaction had been noted, ascorbate was proposed to be oxidized by O_2_ as part of a respiratory chain, possibly linked to ascorbate oxidase (Fig. 1a).

The discovery of superoxide dismutase in mammals and then in plants underlined the physiological importance of oxygen free radicals in biological cells (McCord and Fridovich 1969; Sawada et al. 1972; Asada et al. 1973). At the same time, there was an increasing focus on the potentially harmful effects of lipid peroxidation (Heath and Packer 1968), growing awareness that optimal activities of photosynthetic enzymes required a highly reducing environment (Wolosiuk and Buchanan 1977; Charles and Halliwell 1980), and interest in the mechanisms of ‘pseudocyclic electron transport’ in the chloroplast. The last involves oxygen reduction and is a process that can act to balance chloroplast ATP:NADPH ratios or function as an alternative sink for electrons but which also generates ROS as intermediates (Allen and Hall 1974; Asada et al. 1974). Developments such as these spurred interest in the reactions by which oxidizing ROS could be prevented from accumulating to detrimental levels in the illuminated chloroplast.

It was within this context that a paper in Planta (Foyer and Halliwell 1976) proposed a chloroplast pathway for removal of superoxide and H_2_O_2_ (Fig. 1b). In retrospect, the article can be seen as one of the foundation stones of what is now often called the ascorbate–glutathione pathway, or Foyer–Halliwell–Asada pathway (Foyer and Kunert 2024). This perspective takes a look back at the significance of this work in building knowledge about this area of antioxidant metabolism in plants and some of the developments in the field since.

## The H_2_O_2_-ascorbate–glutathione pathway takes shape in the chloroplast

Within the context of concepts of the importance of ascorbate and glutathione in plants, the study of Foyer and Halliwell (1976) reported two notable advances. First, it provided new information on the subcellular compartmentation of glutathione and GR. By exploiting improvements in techniques for the isolation of ‘intact’ spinach chloroplasts that retain the chloroplast envelope and therefore stromal components (as well as the thylakoid membranes), the authors were able to locate appreciable GR activity and contents of glutathione in this organelle. Second, the study proposed a simple metabolic scheme that turned out to be very influential in promoting further studies in the area of ROS metabolism (Fig. 1b). At a time when catalase was the best-known H_2_O_2_-removing enzyme in plants, the paper emphasised the integration of ROS removal with NADPH-producing metabolism. The authors noted that, within the context of their focus on the chloroplast, NADPH could be produced from the photosynthetic electron transport chain (thus providing an additional sink for electrons) or, in the dark, by the dehydrogenases of the oxidative pentose phosphate pathway (Foyer and Halliwell 1976). While peroxidases were known at the time, and their possible involvement in ascorbate oxidation previously noted (Mapson and Goddard 1951), the physiological functions of such enzymes were considered to be as much in H_2_O_2_-dependent oxidations as reductant-dependent H_2_O_2_ removal; i.e., they were not necessarily acting primarily as antioxidative enzymes. Moreover, like catalase, their presence in the chloroplast was uncertain. 

At the time of the paper’s publication, the proposed pathway still lacked definition at the enzymatic level. Although a dehydroascorbate reductase (DHAR) activity had long been known to exist in plants, it was found to be low or absent in chloroplasts (Foyer and Halliwell 1976). A partially purified leaf enzyme with DHAR activity was subsequently reported, but was considered likely to be cytosolic (Foyer and Halliwell 1977). Stromal DHAR was thought to be superfluous, given the fast chemical reduction of dehydroascorbate (DHA) by reduced glutathione (GSH), particularly at alkaline pH (Foyer and Halliwell 1976, 1977). Key insights into the link between ascorbate and H_2_O_2_ appeared shortly afterwards, when two independent studies reported ascorbate-specific peroxidase (APX) activities associated with both the thylakoid membrane and the stroma (Groden and Beck 1979; Kelly and Latzko 1979). All of these advances continued to provoke interest in the area, and a few years later an additional chloroplast reductase was characterised. Monodehydroascorbate reductase (MDHAR) activity had been previously detected in plant tissues but the study of Hossain et al. (1984) showed that, like APX and glutathione reductase (GR), it was in located in chloroplasts and, like GR, a flavoprotein. Thus, MDHAR allowed an additional input of reductant to the ascorbate-dependent removal of H_2_O_2_. Unlike the NADPH-specific GR, MDHAR can use NADH or NADPH (Hossain et al. 1984).

As understood today, the ascorbate–glutathione pathway involves four enzymes (Fig. 1c). The pathway is sometimes referred to as a cycle, but according to its classical representation, it is a linear sequence that links reduction of H_2_O_2_, catalysed by APX, to oxidation of NADH or NADPH, catalysed by MDHAR and GR (Fig. 1c). Any cycling refers to the ascorbate and glutathione pools, which are interconverted between the oxidized and reduced forms. Although Fig. 1c emphasises APX-dependent removal of H_2_O_2_, other ROS such as superoxide may also chemically oxidize both ascorbate and glutathione (Polle 2001).

## The ascorbate–glutathione pathway: new subcellular horizons

The early work on redox exchange between ascorbate and glutathione had investigated extracts of whole tissues. Cellular and subcellular compartmentation was yet to be elucidated. Much of the research on ROS production and redox regulation in the 1970s and 1980s was focused on the chloroplast, and the paper by Foyer and Halliwell (1976) shone a light firmly on ascorbate and glutathione specifically within this compartment. Subsequently, however, the different enzymes were also found to be present at other subcellular locations. Isoforms of APX and GR were characterized in the cytosol and elsewhere in the cell (Edwards et al. 1990; Mittler and Zilinskas 1991). Despite their high concentration of catalase, peroxisomes were found to house most components of the pathway (Jiménez et al. 1997). A pea gene was reported that encoded a GR found not only in the chloroplast but also the mitochondrion (Creissen et al. 1995). With the rise of Arabidopsis as a study system, and notably genome sequencing, the pathway and its genes were characterized at the molecular genetic level, revealing (or confirming) that all four enzymes were encoded by more than one gene, thus explaining the presence of the enzyme activities in several compartments (Table 1).

**Table 1:** The four enzymes of the ascorbate–glutathione pathway

Enzyme	Name	Gene code	Principal subcellular compartment(s)
APX	APX1	At1g07890	Cytosol
	APX2	At3g09640	Cytosol
	APX3	At4g35000	Peroxisomal membrane
	APX4	At4g09010	Peroxisome matrix(?), Intrathylakoid space(?)
	APX5	At4g35970	Peroxisomal membrane
	APX6	At4g32320	Cytosol
	sAPX	At4g08390	Chloroplast stroma, Mitochondrion
	tAPX	At1g77490	Chloroplast thylakoid membrane-associated
MDHAR	MDHAR1	At3g52880	Peroxisomal matrix
	MDHAR2	At5g03630	Cytosol
	MDHAR3	At3g09940	Cytosol
	MDHAR4	At3g27820	Peroxisomal membrane
	MDHAR5/6	At1g63940	Chloroplast, Mitochondrion
DHAR	DHAR1	At1g19570	Cytosol, Peroxisome(?), Mitochondrion(?)
	DHAR2	At1g75270	Cytosol
	DHAR3	At5g16710	Chloroplast
GR	GR1	At3g24170	Cytosol, Peroxisome
	GR2	At3g54660	Chloroplast, Mitochondrion

Genes encoding isoforms located in multiple compartments in Arabidopsis

APX, ascorbate peroxidase. DHAR, dehydroascorbate reductase. GR, glutathione reductase. MDHAR, monodehydroascorbate reductase. Only the principal subcellular locations are shown, and are assigned based on database information and the following publications: Chew et al. (2003), Davletova et al. (2005), Lisenbee et al. (2005), Narendra et al. (2006), Kataya and Reumann (2010), Rahantaniana et al. (2017), Gollan et al. (2021). Other enzymes may contribute to some of the activities, and certain isoforms may also be partly located in other compartments such as the nucleus (e.g., GR1). The catalytic sites of some forms located on the peroxisomal membrane may be oriented to the cytosolic side (e.g., APX3, MDHAR4) while the precise enzymatic activity is not yet clear for some of the listed proteins (e.g., APX4, DHAR2)

Although the enzymes of the pathway are found at multiple locations within the cell, there is as yet little convincing evidence that they operate in the apoplastic space (Foyer and Kunert 2024). Literature reports of extracellular activity exist in various species, but genes that direct the enzymes to this compartment remain to be identified. Many reports that detect activities in the apoplast might be largely documenting contamination from the cytoplasm, either during sample preparation and/or as a result of cell disruption caused by stress treatments. While the latter might be relevant to conditions such as pathogen challenge, current concepts suggest that the relatively low antioxidant complement of the apoplast enables this compartment to exist in a more oxidized state compared to the cell interior (Pignocchi et al. 2006). This could be crucial to allow extracellular ROS to accumulate in response to pathogens or during long-range cell-to-cell signalling (Miller et al. 2009). Such accumulation also promotes cell wall metabolism and cross-linking through oxidative processes.

Along with the apoplast, some intracellular compartments might also be deficient in the ascorbate–glutathione pathway, in part to allow them to function in a more oxidizing environment. Examples are the thylakoid lumen, the interior of the endoplasmic reticulum, and the vacuole. It is notable that even if the pathway is absent, ascorbate is present in some of these compartments, albeit in more oxidised form (Conklin et al. 2024; Foyer and Kunert 2024).

## Genetic redundancy within the pathway

Given the genetic complexity of the pathway (Table 1), gene-specific Arabidopsis mutants have been very useful in defining the importance of the different isoforms. While some of the mutants show phenotypic alterations, it is striking how many of them seem to be dispensable for Arabidopsis development in standard conditions. Explanations could include in-built redundancy between the multiple isoforms, the presence of other antioxidant systems (see below), and the ability of H_2_O_2_ or pathway intermediates to move between compartments (Davletova et al. 2005). The possibility of redundancy between MDHARs and DHARs is evident, since they represent alternative modes of ascorbate regeneration (Fig. 1c). Of the different genes encoding the four enzymes of the pathway, only knockout mutants for *GR2*, encoding the dual-addressed chloroplast/mitochondrial GR, have been found be lethal, with this effect being linked to a chloroplast function (Marty et al. 2019). It remains unclear whether this effect relates to the role of the enzyme within the ascorbate–glutathione pathway or to other processes that require chloroplast GR.

## Operation of the pathway in vivo: unresolved issues

The ascorbate–glutathione pathway as understood today does not easily lend itself to the use of nuclear isotopes to estimate metabolic flux between the different reactions. Hence, the system is not amenable to analysis in the same way as some pathways of carbon, nitrogen, and sulphur metabolism. So how representative is the now-accepted scheme shown in Fig. 1c of how the pathway works in vivo? It is clear from the studies going back many decades that ascorbate and glutathione can be redox-coupled. But to what extent does this occur in the plant? And to what extent do the supporting reductants (GSH, NADH, NADPH) contribute to regenerating ascorbate that is oxidized by H_2_O_2_ or other molecules? Evidence that the pathway can work in an integrated fashion came from the effects of added oxidants on ascorbate and glutathione in purified chloroplasts (Law et al. 1983). Other indirect evidence also comes from the coordinated response of enzyme activities and corresponding gene expression that is often observed in response to stress. However, some issues remain to be resolved, and a few of these are now briefly considered.

## MDHAR or DHAR: which is more important in keeping ascorbate reduced?

**Fig. 2:**
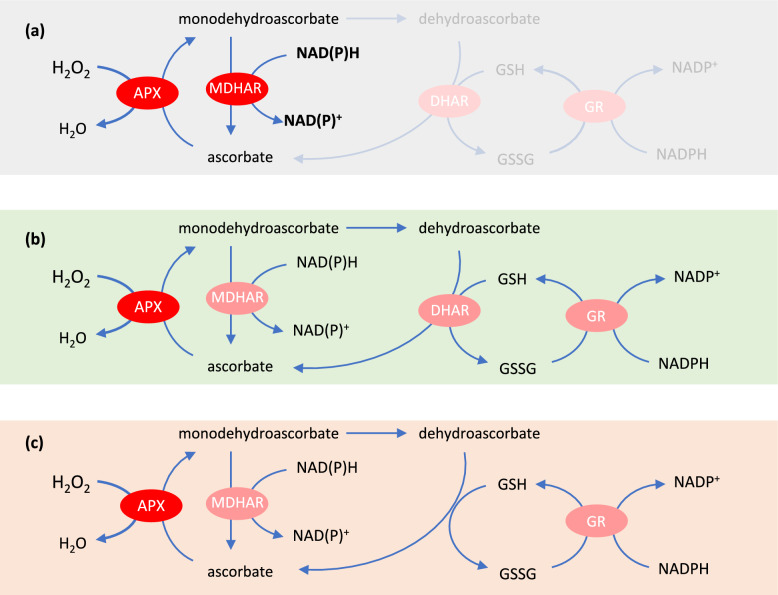
Possible different operating modes of the pathway. **a** MDHAR operating to recycle ascorbate, independent of DHA, glutathione, and GR. **b** MDHAR and DHAR working together, with the latter relying on the glutathione/GR system. **c** As in **b** but without DHAR: reduction of DHA by GSH is purely chemical. See text for further discussion

In many tissues, oxidized forms of ascorbate are present at low levels, with the pool being typically > 80% reduced. Further, several studies have shown that leaf ascorbate can remain in this highly reduced state, even in conditions where oxidative stress is clearly occurring and glutathione is oxidized. While this might partly subcellular compartmentation and the lower reducing power (higher redox potential) of ascorbate compared to glutathione, NADH, and NADPH, it also points to efficient co-operation between the various isoforms of the three reductases. The presence of MDHAR, if it is effective enough in competing with MDHA dismutation, means that DHAR, glutathione, and GR are not necessarily required to regenerate ascorbate (Fig. 2a). However, because some MDHA will inevitably escape reduction and undergo dismutation, a backup system to deal with DHA is necessary (Fig. 2b, c). But how much traffic does this backup system have to deal with?

The relative extractable activities of the three reductases are often lower than that of APX, and this is particularly the case for GR. For example, total leaf APX activities in Arabidopsis leaves are five to ten times greater than GR activities (e.g., Rahantaniana et al. 2017). While this offers little hard information on flux through the various steps, it is consistent with glutathione making a relatively small contribution to ascorbate regeneration, all the more so if GR also has to maintain GSH oxidized by DHA-independent processes. In agreement with this, kinetic modelling suggests that MDHAR will be the predominant contributor to ascorbate regeneration (Polle 2001). This might especially be the case at low rates of ascorbate oxidation, which will probably entail low accumulation of MDHA and low rates of DHA formation (Tuzet et al. 2019). In such conditions, the APX-MDHAR system could work largely as a closed cycle, with minimal or minor input from glutathione (Fig. 2a). When flux is increased, the glutathione/GR system is likely to be increasingly solicited to provide additional support for ascorbate regeneration (Tuzet et al. 2019), or to support regeneration of GSH oxidized by other compounds such as superoxide (Polle 2001), as well as some of the thiol peroxidases discussed further below.

The picture is complicated by the presence of additional MDHA- and DHA-reducing systems, in addition to the enzymes encoded by the genes shown in Table 1. While five genes are annotated to encode MDHAR in Arabidopsis, several other routes might catalyse MDHA reduction. In addition to ferredoxin in the chloroplast (Miyake and Asada 1994), MDHA-reducing activities have been described for a protein located at the plasma membrane and a tonoplast cytochrome *b* enzyme (Bérczi and Møller 1998; Gradogna et al. 2023). MDHA reduction by a 12-oxo-phytodieonic acid reductase has also been documented (Maynard et al. 2020). In the case of DHA reduction, several types of protein can contribute (Foyer and Mullineaux 1998). It is likely that many such systems have relatively low capacities, although they may play crucial site-specific or conditional roles.

## DHA reduction by GSH: enzymatic or not?

Single, double and triple knockout mutants for DHAR have been reported (Noshi et al. 2017; Rahantaniana et al. 2017; Terai et al. 2020; Hamada et al. 2023). While marked phenotypes have not been reported for these systems, they have been used to try to establish the importance of the enzymes. This issue is of particular interest because simple in vitro experiments have shown that GSH can rapidly reduce DHA, even in the absence of an enzyme (Fig. 2c). So how important to the plant are enzymes with DHAR activity? The presence of these enzymes seems in itself to be good evidence that they are somehow required to optimise GSH-dependent reduction of DHA, although other functions cannot be ruled out.

As noted by Foyer and Halliwell (1976), the chemical reaction between DHA and GSH is much more rapid at higher values of pH (about tenfold faster at pH 8 than pH 7; Winkler et al. 1994). Hence, DHARs might play a more important role in the cytosol than in the chloroplast stroma, where the pH is about 8 in the light. This is consistent with the abolition of most of the H_2_O_2_-induced glutathione oxidation in mutants for the cytosolic isoforms (Rahantaniana et al. 2017). However, this observation is somewhat difficult to reconcile with modelled data (Polle 2001). Even using the lower rate constants for the chemical reaction in models, DHAR appears to be dispensable (Tuzet et al. 2019; authors’ unpublished data).

In the chloroplast, high light should promote significant alkalinization of the stroma and therefore favour the chemical reaction. Nevertheless, evidence was obtained that the enzymatic reaction is also required to sustain ascorbate accumulation induced in these conditions (Hamada et al. 2023). Further work is required to elucidate the significance of DHAR activity and to clarify discrepancies between modelled and experimental data. Two possibilities are that DHA concentrations are extremely low in compartments such as the chloroplast and cytosol or that rate constants for the chemical reaction are much slower in the crowded cellular environment than those measured in dilute solutions in vitro, making enzymes necessary to favour the reaction between DHA and GSH.

## Interaction with other ROS-processing pathways

**Fig. 3:**
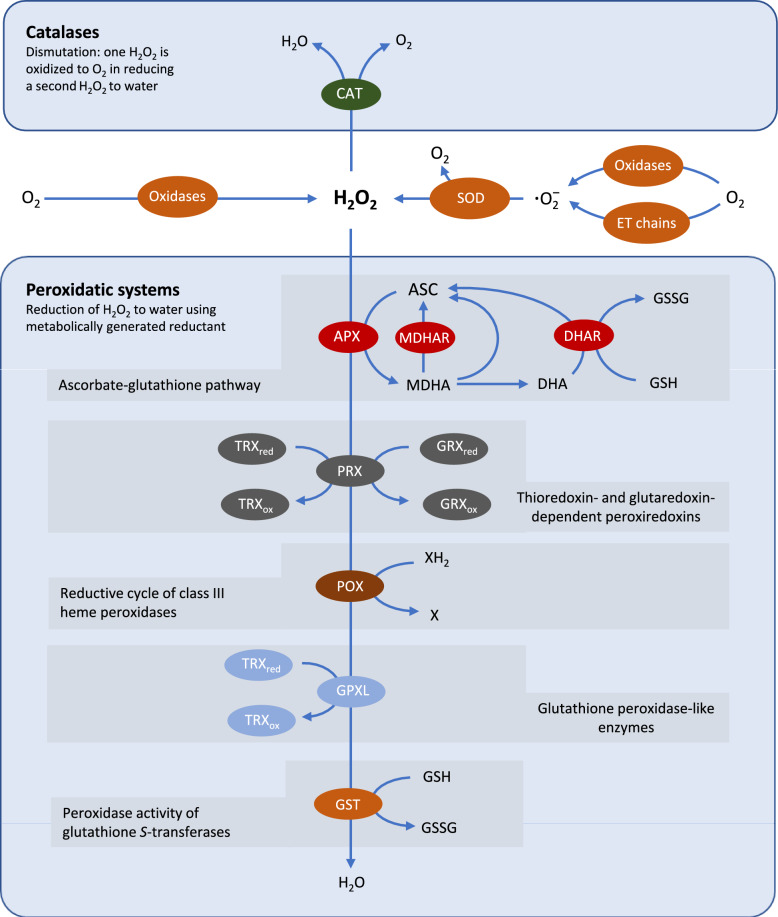
Keeping H_2_O_2_ concentrations down: the complexity of plant antioxidative systems. ASC, reduced ascorbate. CAT, catalase. DHA(R), dehydroascorbate (reductase). ET, electron transport. GPXL, glutathione peroxidase-like enzyme. GSH, reduced glutathione. GR, glutathione reductase. GRX, glutaredoxin. GSSG, glutathione disulfide. GST, glutathione *S*-transferase. MDHA(R), monodehydroascorbate (reductase). Ox, oxidized. POX, class III heme peroxidases (guaiacol-type peroxidase). PRX, peroxiredoxin. Red, reduced. SOD, superoxide dismutase. TRX, thioredoxin. X/XH_2_, oxidized and reduced forms of POX electron donors. The scheme is not meant to an exhaustive representation of reactions that can remove H_2_O_2_. Reactions are not shown stoichiometrically and not all co-factors are shown. Other donors such as ascorbate and glutathione may function with certain PRX

Since the elucidation of the ascorbate–glutathione pathway, it has become apparent that plants have multiple pathways for ROS removal. In particular, several other types of peroxidases have been characterized that can reduce H_2_O_2_ to water (Fig. 3). Class III heme peroxidases (POX, also sometimes called ‘POD’) are activities that have been studied in plants from long before the discovery of APX, and are now known to be encoded by numerous genes (> 70 in Arabidopsis). The combined activity of these enzyme is often used as a marker for oxidative stress but they can also have pro-oxidant roles (O’Brien et al. 2012). Even when acting to reduce H_2_O_2_, their most important function may be in oxidizing the co-substrate rather than removing ROS (O’Brien et al. 2012). Glutathione *S*-transferases (GSTs) form another large gene family, with > 50 members in Arabidopsis. As well as the three DHARs listed in Table 1, the GST family includes several proteins with GSH peroxidase activity (Dixon et al. 2009). Like some POX, certain GSTs are strongly induced by oxidative stress, although it remains unclear whether this is related to peroxidase activities or rather conjugation of stress-induced metabolites and hormones (Lelarge-Trouverie et al. 2023).

Two classes of thiol peroxidases were first reported in plants in the 1990s: peroxiredoxins (PRX) and the so-called glutathione peroxidases (GPX) (Holland et al. 1993; Baier and Dietz 1997; Eshdat et al. 1997). The PRX gene family encodes several types that are regenerated by different reductants, notably thioredoxins or glutaredoxins (Baier and Dietz 1997; Rouhier et al. 2002). Along with GSTs, some of these enzymes offer additional routes by which ascorbate and, particularly, glutathione can be linked to peroxide metabolism. Despite the current nomenclature, plant GPXs are not likely to be highly dependent on the glutathione pool. While these proteins are important antioxidative enzymes in mammals, the plant forms have a different catalytic mechanism and several independent studies strongly suggest that they preferentially use thioredoxins as reductant (Herbette et al. 2002; Iqbal et al. 2006; Navrot et al. 2006). They are probably misleadingly named and might be better termed GPX-like (Fig. 3).

Why so many different pathways to metabolize H_2_O_2_ in plants? Apart from differential compartmentation, one possibility is that this array of enzymes guarantees robust redox homeostasis, even at the expense of redundancy, consistent with loss-of-function studies for APX and PRX isoforms in the chloroplast (Awad et al. 2015). An important distinction is oxidant specificity. While catalase and APX have a strong preference for H_2_O_2_, many of the other peroxidases are also able to metabolise organic peroxides (Eshdat et al. 1997; Dixon et al. 2009). Among other issues are differences in capacity and affinity for the oxidizing substrate, a potential role of thiol peroxidases in H_2_O_2_ perception and, as mentioned above, the importance of other peroxidases in H_2_O_2_-dependent substrate oxidations or ROS generation.

## Ascorbate and glutathione in the age of ROS signalling

The paper by Foyer and Halliwell (1976) was published at a time when most of the attention paid to ROS was either as intermediates in the regulation of electron transport or in secondary metabolism. Neither of these roles has been disproved, but interest in them became somewhat overshadowed by conceptual developments in other directions. With the increasing focus of research on stress in plants that developed in the 1980s, ROS came to be seen as inevitable molecular thugs, a price that had to be paid for aerobic metabolism and that could best be met by careful policing via antioxidant systems. This view motivated attempts to confer stress resistance in plants by overexpression of antioxidative enzymes (eg, Foyer et al. 1995). Although such projects continue today, several discoveries led to the widespread acceptance that ROS are an integral and necessary part of plant metabolism. Most notably, it was shown that ROS are not only incidental side-products of metabolism but also generated by systems that have evolved to do so in a ‘programmed’ manner (Doke 1985; Desikan et al. 1996; Foreman et al. 2003; Kwak et al. 2003; Torres et al. 2002). These and other findings have led to the currently predominant view of ROS as important signaling molecules. Within this context, the ascorbate–glutathione pathway is a key part of the nexus that acts to dampen excessive fluctuations in H_2_O_2_ concentrations (Fig. 3). Here, it is worth noting that APX is less phylogenetically widespread than some antioxidative enzymes, and is largely specific to photosynthetic organisms. This possibly reflects the abundance of ascorbate and the high rates of H_2_O_2_ production in plants. In organisms that lack APX, ascorbate is likely to be a much less important player in H_2_O_2_ removal, since the chemical reaction between the two compounds is slow.

Perturbations of metabolic status within the pathway as ROS-processing activity increases could contribute to oxidant-triggered signalling. Several mechanisms are possible, including pro-oxidant roles of certain enzymes in specific biochemical contexts (Johnston et al. 2015). Under conditions of increased oxidative load in the cytosol, glutathione oxidation is favoured by DHAR and antagonised by MDHAR (Rahantaniana et al. 2017; Xu et al. 2025), providing evidence that ascorbate and glutathione pools are redox-coupled in vivo and that the pathway can operate in different modes depending on flux and the relative capacities of these two reductases (Fig. 2a, b). Whatever share of the flux calls on GSH, DHA-dependent changes in the status of glutathione and related molecules such as *S*-nitrosoglutathione (GSNO) might be detected by the cell as a readout of increased oxidative load (Lindermayr 2018; Zhang et al. 2020). The possibility that oxidized forms of ascorbate play signalling roles cannot be discounted (Xu et al. 2025), although mechanisms are not yet well defined. Evidence that the enzymes themselves can be modified by redox-related processes such as *S*-nitrosylation (Romero-Puertas and Sandalio 2016), in addition to the transcriptional control of the genes encoding them, emphasizes the intricacy of the checks and balances operating to maintain redox homeostasis within the cellular environment.

## Concluding remarks

Many of the foundational studies of the ascorbate–glutathione pathway in the 1970s and 1980s were conducted in plant species such as spinach and pea. Subsequently, the availability of incisive tools and other features have made Arabidopsis a model plant for investigating redox functions *in planta*. While research on crop species is being increasingly promoted, redox interactions are fundamental to the development and acclimation of all plants, and their elucidation is likely to be favoured by continued research on model systems. Thanks to such research, we have an ever-growing appreciation of the complexity and importance of the redox-homeostatic systems that are required for plant growth and survival. Loss-of-function Arabidopsis mutants have been particularly useful. To date, most components have been found not to be essential for plant development, at least under standard growth conditions. This probably reflects the robustness and redundancy of plant antioxidative systems. Given the title of the paper by Foyer and Halliwell (1976), it is intriguing that notable exceptions to this redundancy are glutathione synthesis, ascorbate synthesis, and chloroplastic GR activity (Cairns et al. 2006; Dowdle et al. 2007; Marty et al. 2019). While this might be largely coincidental, current concepts suggest that the co-operative roles of ascorbate and glutathione in the Foyer–Halliwell–Asada pathway are among the most important pillars of the antioxidant network. 

## Data Availability

The manuscript contains no data.
